# Supplementary material for: Are carbon emissions trading and green financial instruments synergistic? -Comprehensive quantitative research based on content analysis
Source: PLoS One. 2024 Mar 7;19(3):e0298601. doi: 10.1371/journal.pone.0298601 (PMC10919601; doi:10.1371/journal.pone.0298601)
Supplement: S1 Data — (ZIP) [file pone.0298601.s001.zip › Data/地级市Z_F工作报告.zip!/─■╧─/╬Γ╓╥╩╨/└┤╫╘╣1⁄2╓┌║┼í╛┬φ┐╦╩2╛▌═°í┐_2015.docx]

各位代表：

现在，我代表市人民政府向大会报告政府工作，请予审议，并请市政协委员和其他列席人员提出意见。

一、勇于担当，砥砺前行，经济社会实现平稳健康发展

过去的一年，面对复杂严峻的经济形势和繁重艰巨的发展任务，在自治区党委、政府和市委的正确领导下，在市人大、政协的有力支持下，全市上下紧紧围绕“改革开放、转型发展”主题，以“两场赛跑”为抓手，攻坚克难，狠抓落实，全力做好稳增长、转方式、促改革、惠民生等各项工作，较好地完成了市四届人大三次会议确定的各项任务。

（一）在经济下行压力持续加大的不利形势下，我们综合施策、精准发力，经济运行实现稳中有进。去年上半年我市几项主要经济指标增幅位居全区前列，进入第三季度，经济运行出现波动和异常。面对严峻形势，我们坚持问题导向，科学研判，沉着应对，有效扭转了下滑势头，经济实现了企稳回升。全市完成地区生产总值383.4亿元，增长7.6%；地方公共财政预算收入35.1亿元，可比增长15%；社会消费品零售总额87.6亿元，增长12%；城镇居民人均可支配收入19853元，增长8.5%；农村常住居民人均可支配收入8442元，增长11%。一是强化服务保运行。全面落实停产企业帮扶、重点项目包抓、定期监测调度等措施，兑现扶持资金1亿元，减免行政性收费1500万元，降低用电、物流等生产成本4200万元，及时协调解决企业恢复生产、市场销售等问题。从力所能及的一点一滴做起，帮助企业坚定信心，渡过难关。规模以上工业实现增加值149.9亿元，增长13.2%，增速居五市第一，高于全区5个百分点。二是拓展渠道抓融资。出台金融改革创新意见，帮助企业搭建融资平台，引来源头活水。12家企业上市融资，新设立9家金融分支机构，中小微企业新增贷款20亿元，融资难、贷款难的问题得到有效缓解。我市被确定为全国31个小微企业信用体系建设试验区之一。三是投资拉动促增长。越是压力增大，越是狠抓“两大任务”不放松。全年落实上争项目1541个，落实批复资金123.8亿元。实施招商引资项目456个，到位资金538亿元。年初确定的180个重点项目全部开工，普华新型合金材料、宁夏伊佳服饰等50个项目建成投产，市医院迁建、吴忠热电厂等重大项目顺利推进。全市完成社会固定资产投资602.1亿元，增长21.2%。

（二）在既保增长又调结构的双重压力下，我们量质并举、增调同步，转型发展迈出坚实步伐。通过政策引导、市场倒逼等有效手段，加大结构调整力度，推动产业优化升级，促进经济提质增效。工业转型步伐加快。新增规模以上工业企业84家，亿元企业14家。坚持抓优势产业扩增量，金积工业园区产值突破百亿元，新能源装机容量达到360万千瓦，占全区60%以上，轻重工业比达到29:71，轻工业占比高于全区14.8个百分点。促成华盈镁业与中煤华昱、涝河桥食品公司与华澳集团合作发展，有效盘活了存量、扩大了增量。实施技改项目30个，坚决淘汰落后产能100.8万吨，单位工业增加值能耗下降14%，万元GDP能耗下降6%，超额完成了自治区下达的节能目标。现代农业活力增强。粮食产量实现“十一连增”。建成恒源、上陵等千头以上牧场21个，全市奶牛存栏20.3万头，肉牛、羊饲养量分别达到55万头和600万只，特色优势产业产值占农业总产值的90%以上。新增亿嘉甘草、禹皇葡萄酒等龙头企业23家，培育新型合作组织110家，流转土地80万亩。农田水利基本建设勇夺全区“黄河杯”八连冠。第三产业稳步发展。市区“十大市场” 和7个县级专业市场投入运营。农超对接“直采直供”模式在全国推广，电子商务初显端倪。开通宁波-吴忠旅游包机航线，秦坝关村等5个村庄入选首批中国少数民族特色村寨，全年接待游客286万人次，旅游收入15.5亿元，分别增长28.2%和20%。金融业运行平稳，存贷款余额分别增长9%和12.8%。房地产业完成投资70亿元，增长25%。

（三）在山川、城乡发展差距仍然较大的实际情况下，我们统筹兼顾、协调推进，城乡一体化进程不断加快。实施城乡建设项目180个，完成投资152.3亿元，城镇化率达到53.3%。文体会展中心、乃光湖公园等33个沿黄城市带重点建设项目顺利推进。完成30公里供热管网改造，打通市区断头路25条22公里，改造老旧小区11个，实施既有建筑节能改造110万平方米。红寺堡政务服务中心、图书馆建成使用，盐池群众艺术馆、同心“三馆一中心”等重点项目深入推进。新建美丽小城镇6个，美丽村庄示范点30个，建设农村公路608公里。深入开展主干道路大整治大绿化活动，城乡环境不断改善。完成造林39.9万亩，全市森林面积436万亩，森林覆盖率达到14%，治理水土流失面积280平方公里。二氧化硫等四项主要污染物减排任务全面完成。我市荣获“中国人居环境范例奖”。

（四）在深化改革开放面临诸多瓶颈制约下，我们大胆探索、重点突破，发展活力进一步彰显。205项改革任务完成188项，启动改革试点12项。市县政府机构改革基本完成。行政审批事项、办结时限、审批要件分别压减72%、63%、21%。全面推行“项目四级服务制”、“五个一”审批模式，建成“三位一体”服务平台，开通“51890”便民热线。在服务业实行“先业次照再证后规范”工商注册程序，取消中小微企业注册资本下限，全市新登记中小微企业2733户，市场主体总量达到7.23万户，增长97%，居五市第二。科技创新步伐加快，吴忠仪表高端控制阀关键技术自主创新和产业化项目获国家科技进步二等奖，青铝集团等3家企业被列为国家级“两化融合”标杆示范企业。联合国内35家知名企业共同组建新丝路产业联盟。全市外贸进出口总额突破1亿美元，增长10%。

（五）在财政收支矛盾不断加剧的困难局面下，我们坚持底线思维、优先保障民生，让人民群众享受更多发展成果。民生事业支出126亿元，占公共财政支出的87.5%。承诺为民办理的11件实事全面完成。大力实施精准扶贫，整合投入各类资金9.1亿元，3个整乡推进试点和100个整村推进项目进展顺利，20个村脱贫销号。新建续建移民安置房3470套，搬迁安置移民1.57万人，青铜峡、盐池、同心全面完成“十二五”县内移民任务。建设保障性住房11978套，改造农村危房5454户，帮助一大批困难家庭圆了住房梦。统筹推进城乡居民基本医疗、养老保险，城乡居民基础养老金提高至105元。3.9万城乡居民享受免费普惠性健康体检。市财政从去年开始每年安排2亿元，妥善解决利通区被征地农民养老保险问题。加大教育投入，新建改建中小学校30所、幼儿园16所。利通区、青铜峡市顺利通过国家义务教育均衡发展评估验收。创建国家卫生城市工作扎实推进。364个新建标准化村卫生室全部投入使用，四级医疗卫生服务体系基本健全。全市人口出生率11.98‰。广泛开展“滨河大舞台”等各类文体活动，吴忠文化馆被评为全国优秀文化馆。大力发展慈善事业，我市再获“七星级慈善城市”称号，位列“中国城市公益慈善百强榜”第28位。第三次全国经济普查圆满完成，我市荣获全区先进集体。国防教育、人民防空、民兵预备役和双拥工作不断加强，老年人、残疾人、妇女儿童权益得到切实保障，外事侨务、气象、档案、住房公积金等工作取得新成绩。

（六）在转作风抓发展对政府履职尽责的严格要求下，我们狠抓依法行政、创新社会治理，和谐稳定局面进一步巩固。健全重大决策社会稳定风险评估机制，注重从源头预防和化解社会矛盾，妥善化解重点信访积案119件。“六五”普法深入开展，人民调解 “以案定补”、“以奖代补”制度在全区推广。深化平安吴忠建设，刑事案件破案率居五市第一。新一轮禁毒人民战争工作经验在全区推广。安全生产形势总体稳定，应急管理和防震减灾工作不断加强。民族团结进步创建工作持续深化，“385”创建模式得到国家民委高度评价。利通区、同心县被授予全国民族团结进步模范县。扎实开展群众路线教育实践活动，着力解决人民群众反映强烈的突出问题。全市性考核评比减少83.4%，腾退办公用房4万平方米，政府采购累计节约财政资金3127万元。深入开展精神文明创建活动，刘志海等3人荣登“中国好人榜”，赵峰等3人荣获“感动宁夏年度人物”。组织领导干部到自治区廉政警示教育中心接受警示教育。强化行政监察和审计监督，设立纪检、检察视频专线，实现检察院、监察局和公共资源交易中心“三网合一”。高度重视并自觉接受人大法律监督和政协民主监督，认真听取各民主党派、工商联、无党派人士意见建议，办理人大代表议案建议87件、政协委员提案212件，取得良好效果。

各位代表，一份耕耘，一份收获。回顾过去一年的工作，我们顶住了压力，经受住了考验，成绩来之不易。各级干部勤勉敬业、真抓实干，蛮拼的精神值得肯定；广大群众拥护支持、理解配合，朴实的情怀值得点赞。在此，我代表市人民政府，向全市广大工人、农民、知识分子、干部职工，向人大代表、政协委员、民主党派、工商联、人民团体、离退休老同志和社会各界人士，向驻吴人民解放军、武警官兵、政法干警，向中央及区属驻吴单位，表示崇高的敬意和诚挚的感谢！

在肯定成绩的同时，我们清醒地认识到，发展中还存在一些不容忽视的困难和问题。主要表现在：受经济下行大环境和“不唯GDP论”的影响，个别预期经济目标未能如期完成；发展基础还不稳固，资源环境等要素制约日益突出，面临着稳增长与调结构、转方式的双重压力；山川城乡发展差距仍然较大，扶贫攻坚任务十分艰巨，民生改善与群众的期望还有较大差距；骨干财源、优质财源少，民生保障、政府债务等刚性支出加大，财政收支矛盾进一步凸显；发展环境还需进一步优化，效率不高、落实不力、服务不优的问题依然存在，政府自身建设还需不断强化等。这些问题，我们必须高度重视，认真加以解决。

二、主动适应新常态，奋力开创“五个吴忠”建设新局面

新常态是中央着眼当前国际国内经济形势，对我国经济发展阶段性特征的准确判断和深刻把握。市委四届七次全体会议紧紧围绕我市实际，深刻分析阐述了新常态下我市经济发展的特征和要求、机遇和挑战，经济增长的基础和支撑、后劲和动力。我们要牢牢把握和适应经济新常态，把各项工作置于新常态这个大逻辑下来理解、考虑和谋划，把新常态的要求贯彻到思想理念、发展方式、工作方法、作风建设等各个方面，做到观念上适应、认识上到位、方法上对路、措施上得力，牢牢掌握工作主动权。

（一）新常态下，更要坚持发展是硬道理的战略思想。中央经济工作会议把“努力保持经济稳定增长”作为今年的首要任务，充分说明新常态下稳增长的力度松不得。市委四届七次全会强调：“我市是欠发达地区，发展是第一要务。不唯GDP不是不要GDP，不论形势如何变化，经济建设是硬道理”。当前，我市面临的最根本问题还是发展不够、发展不足的问题，在条件具备的情况下争取发展得更快一些，这是欠发达阶段的必然要求，也是吴忠最大的实际。没有平稳较快的经济增长做基础，调结构、惠民生、促发展，都将成为“画饼”。我们要进一步强化发展意识，以更大的信心和决心推动新常态下的新发展，在发展问题上任何时候都不能分神、松劲、失速。同时，新常态下，我们必须牢牢把握解放思想这把“金钥匙”，不断寻求思想理念的更新与突破，以思想大解放推动经济社会大发展。

（二）新常态下，更要注重经济发展质量和效益。适应经济新常态，必须摒弃“高投入、高排放、高污染、低效益”的粗放型增长方式，推动经济发展从主要依靠要素投入转换到更多依靠改革创新和结构调整。牢固树立有质量、有效益、可持续的发展意识，坚持做大总量与做优质量并举、培育发展优势产业和改造提升传统产业并推、增加财政收入和提高居民收入并重，推动经济总量不断提升，结构不断优化，内生动力不断增强，实现没有水分，实实在在的发展。

（三）新常态下，更要牢牢把握机遇、沉着应对挑战。对吴忠而言，新常态是挑战，更是机遇。一方面，我市经济下行压力依然较大，固定资产投资增速放缓，新的经济增长点培育不足，改善民生任务繁重，全面小康实现程度仍低于全区平均水平。另一方面，中央继续实施积极的财政政策和稳健的货币政策，进一步加大对西部少数民族地区的倾斜扶持力度；国家“一带一路”发展战略的引领带动，自治区“两区”建设的深入推进，宁夏空间发展战略规划的启动实施，东部地区产业的加速转移；加之近年来全市上下艰苦努力奠定的坚实发展基础、蓄积的强大精神动力，这些都将为我们加快发展提供广阔空间和有力保障。只要我们积极适应新常态，主动应对新常态，完全有条件、有能力实现有质量、有效益的发展。

（四）新常态下，更要坚守民生和生态两条底线。经济为重，民生乃大。伴随着经济发展进入新常态，面对人民过上更好生活的新期待，我们不能有丝毫自满和懈怠，必须再接再厉，抓住群众最关心最直接最现实的利益问题，保住基本、补上短板、兜好底线，解决好衣食住行、教育、医疗、文化、养老、安全等问题，让群众有事干、有钱赚、有盼头，日子越过越好。同样，适应新常态，对推进生态文明建设和环境保护，是一个重大责任。我们要坚守生态、耕地、水资源三条红线，努力做到不欠新账、多还旧账，真正使经济发展代价更小、效益更好、更可持续。

（五）新常态下，更要保持奋发有为的精神状态。做好新常态下的各项工作，必须有推动科学发展、浴火重生的勇气，必须有改革创新、干事创业的担当，必须有求真务实、埋头苦干的劲头。既要克服传统增长模式下以速度为纲的惯性思维，更要走出“速度调低了，就可以少作为、慢作为、甚至是不作为”的认识误区，以攻坚克难、奋发有为的新境界、新作风，奋力开创各项工作新局面。

根据市委四届七次全体会议精神，今年政府工作的总体要求是：认真贯彻党的十八大、十八届三中、四中全会和习近平总书记系列重要讲话以及自治区党委十一届四次、五次全会精神，全面落实市委四届七次全会部署，坚持稳中求进工作总基调，主动适应经济发展新常态，以提高经济发展质量和效益为中心，以“深化改革开放、加快转型发展”为主题，打好固定资产投资、工业转型发展、农业提质增效、三产优化升级“四场硬仗”，加大改革创新，加快城乡统筹，加强生态保护，持续改善民生，强化法治建设，推动“开放、富裕、和谐、美丽、智慧”吴忠建设取得新成效！

主要预期指标：地区生产总值增长8.5%，固定资产投资增长17%以上，公共财政预算收入增长9%左右，社会消费品零售总额增长11%，城镇居民、农村常住居民人均可支配收入分别增长9%、10%以上，城镇登记失业率控制在4%以内，居民消费价格涨幅控制在3.5%以内，完成自治区下达的节能减排任务。

确定上述目标，是我们按照“既要理性、更要积极”的原则，深入分析和研判了今年宏观经济的基本走势和稳增长的总体要求，充分考虑和把握了我市加快发展、调整结构、改善民生的现实需要。实现这些目标，有一定困难和压力，但只要我们保持清醒头脑，善于在变化的形势中捕捉和把握机遇，把形势想得更复杂一点，把困难和挑战估计得更充分一点，把政策措施考虑得更周全一点，坚定信心不动摇，咬定目标不放松，苦干实干不懈怠，经过扎实工作和共同努力，这些目标一定能够实现。

三、以四场硬仗为抓手，全力提升经济发展质量和效益

打好固定资产投资、工业转型发展、农业提质增效、三产优化升级“四场硬仗”是市委四届七次全会做出的重要战略部署。之所以称为“硬仗”，是因为这四方面的工作任务最重、难度最大，其胜败直接事关今年各项目标任务的完成，事关全市长远发展。打好“四场硬仗”的冲锋号已经吹响，我们要以“能打仗、打胜仗”的气魄，科学排兵布阵，统筹有序推进，坚持做到战略自信，战术精准。既要全线出击、全员上阵，又要重点突出、难点突破。

固定资产投资要当先锋、打头阵，打好“持久战”。我市是典型的投资拉动型经济，打好“四场硬仗”，投资是关键。全年确保上争项目资金不低于125亿元，招商引资实际到位资金550亿元。一是紧盯项目开工，建成达效一批好项目。今年全市计划实施七大领域项目740个，年度计划投资980亿元，其中亿元以上的重大项目144个。重点抓好中国自动化产业园、杭萧汽车零部件等产业项目，银西高铁、叶盛黄河公路大桥等基础设施项目，保障性住房、文体会展中心等民生项目。严格落实重点项目包抓责任制和四级服务制，为项目建设提供优质高效服务。已批复或签约的，抓紧做好选址、环评、征地等前期工作，确保早日开工建设；已开工续建的，加快进度，确保早日投产达效。二是紧盯项目引进，签约落实一批新项目。注重招商引资向挑商选资、招才引智转变，通过产业招商、商务招商等方式，千方百计招资金、招技术、招人才。突出招大引强，重点加强与央企、全国500强企业的联系对接，紧盯昊华骏化、台湾晶叶酵素等有投资意向的大企业，争取落地一批投资过10亿元的产业化项目。三是紧抓项目储备，精心编制“十三五”规划。以项目谋划为重点，充分发挥比较优势，紧紧围绕现代能源化工、食品加工业、现代装备制造、生态纺织等优势产业，教育医疗、社会保障、扶贫开发等民生事业，生态保护、大银川都市区建设、城乡统筹等战略部署，做深前期研究，做优总体规划，做精专项规划，高标准、高质量编制好“十三五”规划。密切关注政策动态，重点围绕国家持续加大对西部和民族地区支持、实施“一带一路”、七大项目工程包以及宁夏空间发展战略规划等带来的投资机遇，谋划一批打基础、增后劲、管长远的大项目，力争进入国家、自治区规划大盘子。四是进一步优化投资结构，提高投资质量和效益。在保持投资强度不减的基础上，突出投资重点、改革投资方式、提高投资效率。更加注重产业类投资，以投资结构的优化促进产业结构调整，推动实体经济发展。充分发挥政府投资的引导带动作用，推广政府与社会资本PPP合作模式，鼓励社会资本设立产业和股权投资基金，千方百计创造一切条件让民间资本参与投资，放开经营，放手发展。

工业要挑大梁、做贡献，打好“主攻战”。在“四场硬仗”中工业是主战场、主阵地，必须集中优势兵力，盘活存量、扩大增量、做强总量，充分发挥推动经济增长的主引擎、主力军作用。再增工业发展新动力。实施5000万元以上重点工业项目110个，力促华能大坝2×60万千瓦火电等65个项目开工建设，宁鲁石化60万吨芳烃等45个项目建成投产。力争新增销售收入过亿元企业10家，新增规上企业80家，确保规上工业增加值增长11.5%。进一步优化工业布局，加快“三条工业经济带”建设，集中力量打造10个产值或销售收入过百亿元的产业板块。今年，新材料基地和煤炭电力产业基地产值各突破百亿元，3年内10个产业板块工业总产值突破千亿元。再造特色产业新优势。食品加工、生态纺织、新能源等优势特色产业要扩大规模，尽快形成聚集效应。加快伊利二期、宁夏麒阳等项目建设进度，确保年内建成投产。实施深圳华澳集团食品全产业链项目，深度开发牛羊肉资源，提升产品附加值，打造西北最大的全产业链肉食品加工基地。建立健全食品产品研发监测、仓储物流、展示交易等支撑体系，力争全市食品产业产值增长15%以上。启动恒丰二期高档家纺、服装项目，加速构建上下游产品有序链接、融合配套的纺织产业发展新格局。加快推进中民投光伏发电、运达15万千瓦风电等项目，力争全市风光电装机总容量突破400万千瓦。再添传统产业新活力。重点以15个企业技术创新项目为依托，引导青铝、庆华、华美镁业等能源化工、冶金企业调整产品结构，加快发展下游延伸产品，坚决淘汰昊盛纸业等落后产能，挖掘潜力、降本增效，实现总量增长和结构优化。鼓励通达等企业通过资产收购、兼并重组等多种形式进行整合，实现可持续发展。再施运行监测新举措。进一步强化对工业经济运行的分析与综合协调，加强对全市重点工业产品价格、用电负荷及产品库存监测，及时掌握运行动态，及早提出应对措施。针对一些支撑作用大、拉动效应强的重点企业，采取“点对点”的方式进行帮扶。依托大企业配套发展一批小微企业，降低投资准入门槛，帮助解决好融资、用地、用工等问题，把小微企业扶上马、送一程，让他们更好地成长。加快形成大企业“顶天立地”、小微企业“铺天盖地”的良好发展态势。

农业要提质量、增效益，打好“保卫战”。打好“四场硬仗”，农业要在稳定现有产量、转变发展方式、防御市场风险的基础上提质增效，与二产、三产“联手”，走工业化发展之路，为“四化融合”发展奠定基础。适度扩大规模，提高土地产出率。优质粮食种植面积稳定在100万亩，新增奶牛2万头、肉牛5万头、滩羊20万只，新增枸杞、甘草等中药材种植面积10万亩，瓜菜面积达到52万亩。因地制宜开展农田水利基本建设，实施苦水河治理、扁担沟扬水泵站改造等一批水利工程，新增节水灌溉面积20万亩，治理水土流失面积316平方公里。加大农业科技投入，推广肉羊改良、测土配方施肥等新技术20项，引进新品种30个。主要粮食作物综合机械化水平达到70%以上，科技贡献率达到68%，农业增加值增长5%以上。坚持龙头带动，提升市场竞争力。大力扶持龙头企业，依托农产品加工、物流龙头企业带动优势特色农业产业化发展。鼓励和支持农民自愿将承包土地向专业大户、家庭农场、农民专业合作社、龙头企业流转，年内培育新型经营主体100个。紧盯市场需求，打好特色牌、绿色牌，在特色农产品扩产能、深加工上下功夫，进一步扩大“青铜峡大米”、“盐池滩羊”、“同心有机枸杞”、“孙家滩瓜菜”等具有地理标志产品的影响力。坚持绿色发展，突出生态化保护。充分利用我市土地无污染、风光热充足等自然优势，大力推行农业标准化清洁生产，加强农业面源污染治理，推进农业节能减排，着力发展生态农业。以绿色消费为导向，建立推广农产品质量安全追溯体系，确保“舌尖上的安全”。加快发展集葡萄酒、观光农业、旅游休闲等为一体的生态产业，推出吴忠“紫色名片”。持之以恒地做大吴忠国家农业科技园区，打造生态农业“有机谷”。强化政策扶持，增强抗风险能力。积极探索以农业产业化为纽带、以龙头企业和农民专业合作组织为依托的农业保险发展模式，鼓励和支持保险公司积极开展农业保险业务。完善动物疫病防控等保障服务体系，提高农业安全保障能力。健全鲜奶等农产品市场监测预警机制，有效落实鼓励乳品企业稳定收购鲜奶的补贴政策，设立奶业风险基金，保护奶农利益，确保奶产业持续健康发展。

三产要补短板、出实招，打好“突围战”。三产在我市三次产业中既是重点，又是弱项，必须集中火力求突破，力争第三产业增加值增长7%。传统服务业要挖潜升级。以集聚化、规模化、电商化发展为导向，着力推动“十大市场”繁荣发展，推进中达、欧蓓莎等6个商业综合体投入运营，培育一批年交易额过10亿元的大型市场。坚持房地产市场导向，合理引导住房消费预期，推行建设与租赁并举，通过政府购买方式把符合条件的存量房转成公租房，促进房地产市场平稳发展。文化旅游业要迸发活力。启动实施欢乐谷等文化旅游项目，完善黄河楼、大禹文化园等旅游景点设施。坚持政府引导、企业主体、市场运作方式，加大旅游宣传营销，开发旅游精品线路，打造以黄河文化游、红色经典游、近郊休闲游为主的特色旅游经济圈。力争全年接待游客、旅游总收入均增长20%。新兴服务业要拓展提质。加大扶持引导，鼓励社会资本大力发展家政托幼、健康养老、体育健身等生活性服务业，设计研发、认证咨询、金融保险、会展经济等生产性服务业。培育新型业态，推动电子商务创业孵化基地和“淘宝特色中国吴忠馆”等电商平台建设取得新成果，力争全年电商销售突破1亿元。

四、以产城融合为重点，着力加快新型城镇化进程

抢抓自治区构建“一主三副、两带两轴”战略机遇，加快以城带乡、以工促农、城乡一体、协调发展的新型城镇化进程。

完善规划引领体系。严格遵循《宁夏空间发展战略规划》，统筹生活居住、产业分布、生态建设空间布局，完善城市总体规划、区域性详规和专项规划，着力构建“一区三城、三带三轴、十大升级板块”新型城镇化体系，推进“三规合一”、“多规融合”。加强城乡规划管控，扎实开展违法建设整治活动，切实维护规划的严肃性、科学性和连续性。

提升城市内涵品质。主动融入大银川都市区建设，加快推进银吴同城、吴灵（武）相交、吴永（宁）对接进程，着力打造产业布局合理、互通互联、设施完备、人居环境优美的宜居宜业之地。投资150亿元，实施城乡建设项目150个，重点抓好黄河文化体育会展中心、乃光湖公园、妇幼保健院迁建等工程建设，实施阳光瑞祥、清水湾三期等96万平方米房地产项目，完成如意园等8个老旧小区和50万平方米既有建筑节能改造。新建续建7条城市道路。实施热电厂供热管网、生活垃圾资源化利用发电等基础设施建设工程。

突出产城融合发展。利通区、青铜峡市围绕建设吴忠核心区、银川“后花园”目标，重点发展食品加工业和现代装备制造产业，加快利青一体化建设，做强引领文章。红寺堡区围绕打造“中国优质葡萄酒明星产区”目标，重点发展慈善、葡萄、草畜产业，加快罗山葡萄文化示范园、现代高效节水农业示范基地等项目建设，做实产业文章。盐池县围绕打造“中国森林城市”目标，重点发展油气化工、草畜和生态产业，加快现代草畜产业示范带、城市休闲森林公园等工程建设，做足生态文章。同心县围绕打造“中国西部商贸城市”目标，重点发展民族商贸、羊绒、草畜等产业，实施好民族商贸市场体系、有机枸杞及中药材基地等项目建设，做活特色文章。

加快建设“美丽乡村”。推动公共财政向农村倾斜、公共服务向农村覆盖、社会事业向农村延伸。加快推进现代文明小城镇、中心村和基层村建设。重点打造美丽小城镇5个、美丽村庄20个，改造农村危房3000户，建设农村公路500公里。进一步优化村庄布局，完善设施功能，改善人居环境，培育文明乡风。

加强生态环境保护。大力实施生态立市战略，坚守生态环保底线，打造天蓝地绿、山青水秀、空气清新、环境宜人的靓丽名片。深入推进全国生态文明试点城市和国家环保模范城市创建工作，大力实施移民迁出区生态修复、湿地恢复与保护、城市绿化等生态林业工程，全市营造林28万亩，森林面积达到466万亩，森林覆盖率达到15%。大力推进节电、节水、节材工作，巩固提升“国家可再生能源应用示范城市”成果。着力抓好环境保护“三项行动计划”，下功夫整治大气、化工和重金属等污染，健全环境保护绩效考核、责任追究、社会监督等机制，严格环保准入和污染物排放标准，对环境违法行为“零容忍”。全年环境空气质量优良率力争超过去年水平。

五、以改善民生为根本，努力提升群众幸福指数

牢固树立民生改善投入只增不减，民生实事覆盖面只扩不缩的为民理念，加快完善基本公共服务体系，创新社会治理机制，进一步提升群众幸福指数。

打好扶贫攻坚战富民。坚定不移地实施好生态移民工程，统筹抓好致富产业培育、基础设施完善、社会治理创新等工作，新建续建移民住房1495套，搬迁安置移民6300人，全面完成“十二五”生态移民搬迁任务。严格落实精准扶贫和销号管理机制，整合产业扶持、金融扶贫、闽宁协作等项目和资金，加快建设红寺堡中南部葡萄、同心东部中药材、盐池中部草畜等8个产业扶贫基地，集中力量加大6个整乡、100个整村推进力度，确保年内40个村实现脱贫。

完善社会保障体系惠民。重点做好高校毕业生就业和下岗再就业工作，实行“订单式”培训，转移农村劳动力20万人以上，形成大众创业、万众创新的社会氛围。继续推进被征地农民养老保险工作，启动全民参保登记，扩大“五险”覆盖面，城乡居民养老、医疗保险参保率分别达到90%和98%以上。完善城乡居民大病医疗保险机制，探索城镇职工大病保险途径，实现大病保险全覆盖。完善覆盖城乡居民的社会救助体系，健全临时救助制度，推进城乡低保统筹同标。鼓励社会力量兴办养老机构，健全完善养老服务体系。新建保障性住房7000套，重点解决城乡低收入家庭住房问题。切实维护残疾人的生存权、发展权，保障其平等参与社会生活。开展慈善“十进”和“善行吴忠”系列活动，提升慈善吴忠品牌影响力。

加快发展社会事业利民。巩固扩大义务教育均衡发展成果，实施薄弱学校改造、农村幼儿园建设等工程，新建小学6所、幼儿园5所。整合资源，合理调整学校布局，有效解决“入园难”和“大班额”问题。实施普通高中多元录取政策，推进普通高中多样化发展。扶持发展民办教育，加快现代职业教育体系建设，推进职业教育集团化发展。不断深化医药卫生体制改革，加快市人民医院迁建和利通区人民医院建设步伐，力争年内建成投入使用。扎实推进国家卫生城市创建工作，争取通过国家评估验收。全面落实计生政策，稳定低生育水平，人口出生率控制在14.2‰以内。大力实施文化惠民工程，加强基层综合性文化服务中心建设，广泛开展形式多样的群众性文体活动。积极申报创建第三批国家公共文化服务体系建设示范区，推进基本公共文化服务标准化、均等化、社会化发展。启动全国文明城市创建工作。

创新社会治理安民。加强基层社会服务治理体系建设，坚持实行网格化管理、社会化服务。完善人民调解、行政调解和司法调解联动工作体系，探索网上信访受理，用法治思维和法治方式积极化解矛盾纠纷。深化民族团结进步创建成果，不断提高民族团结进步创建工作规范化、系统化、常态化水平。完善宗教领域矛盾纠纷排查机制，依法打击非法宗教活动，巩固依法管理民族宗教事务成果。深化平安建设，坚决打好新一轮禁毒人民战争，严厉打击多发性侵财等违法犯罪活动。强化食品药品全程监管，抓好安全生产，及时排查和消除各种安全隐患，有效遏制重特大事故发生。加强外事侨务、气象、统计、档案、市志、防震、住房公积金等工作，支持工会、共青团、妇联等群众团体工作，强化国防动员，推进双拥共建，促进军地深度融合发展。

六、以改革创新为突破，不断激发经济社会发展内生动力

坚持把改革创新贯穿于经济社会发展各个领域、各个环节，用改革破解难题、靠创新激发活力、以开放助推发展。

深层次推进重点领域改革。大力推进行政审批制度改革。进一步完善服务规范和标准，在提高效率、规范程序、加强监管、强化服务上下功夫，让部门规范、高效审批，让群众方便、明白办事。加快网上审批系统建设，实行网上申报、网上受理，打造一流的政务服务中心。加快建立覆盖市、县政府的权力清单、责任清单和企业投资负面清单，做到政府部门“法无授权不可为”、“法定职责必须为”，市场主体“法无禁止即可为”。深化推进财税金融改革。加快政府预算“全口径”管理体系建设，强化预算绩效管理。全面公开部门 “三公经费”预决算信息，增强财政透明度。加大综合治税力度，确保应收尽收。继续深化金融改革，构建多层次资本市场体系，拓宽融资渠道。鼓励和引导企业通过股权交易、区域集优债券等方式直接融资，争取年内新增金融分支机构6家，在新三板、股权交易中心挂牌企业15家。稳步推进农村土地产权改革。全面完成农村土地承包经营权、集体建设用地、农民宅基地确权颁证工作。加快农村集体资产股份制改革，开展农村产权抵押融资试点。引导和规范土地经营权有序流转，坚持以粮食和农业为主，避免“非粮化”，坚决禁止耕地“非农化”。鼓励引导企业、社会资金参与水利项目建设、管理、经营，探索跨县（市、区）、行业和用户等多种形式水权转换管理模式。统筹推进社会领域改革。加快推进事业单位分类改革，逐步扩大教育、医疗卫生等事业单位法人结构治理试点范围。加大教育改革力度，试点推行校长职级制，完善教育教学评价体系。探索建立公立医院人事和分配制度改革运行机制。启动户籍制度改革，全面放开落户限制，有序推进农业转移人口市民化。积极推进投资、服务业、能源统计改革。

多举措实施创新驱动战略。强化企业创新主体地位，采取专利资助、科技合作等办法，鼓励和支持企业自主创新、集成创新，建立产学研协同共享的技术创新联盟。助推诚志万胜、吴忠仪表、勤昌轴承等企业将科研成果尽快转化为生产力。积极推进信息化和工业化融合，扎实开展“宽带中国”示范城市和“智慧吴忠”创建工作，抓好合力万兴、圣花米来等信息化项目建设，培育自治区级“两化”融合示范项目10个以上。创新科技特派员服务模式，加快特色食品人才高地、生态纺织产业园人才基地建设，着力培养引进一批创新创业团队、产业领军人才和实用技能人才，建设一批“科技超市”，增强科技创新服务能力。

全方位扩大对外交流合作。主动融入国家“一带一路”建设，积极打造“黄金节点”城市，争取将我市纳入国家和自治区“丝路经济带”建设总体规划，融合推进 “丝路经济带”与内陆开放型经济先行区建设。深化与浙江宁波等沿海地区的互动协作，汇聚新的发展动力。大力发展外向型经济，力争全年外贸进出口总额达到1.2亿美元，增长20%。

七、以依法行政为核心，大力推进法治政府建设

按照职能科学、权责法定、执法严明、公开公正、廉洁高效、守法诚信的法治政府建设新要求，顺应人民对政府工作的新期待，努力以自身建设的新成效提升能力、推动发展、取信于民。

坚持把务实勤政贯穿始终。进一步完善重要工作、重点项目、重大活动领导牵头、部门负责的工作机制，一月一报告，一季度一分析。大力倡导勇于直面矛盾、敢于责任担当的作风，坚决纠正不作为、慢作为、乱作为，引导各级政府工作人员牢固树立责任意识，用实打实的作风干工作，用钉钉子的精神抓落实，持续提高行政执行力、服务力和保障力，形成“九牛爬坡、个个出力”的生动局面。

坚持把依法行政贯穿始终。把依法行政贯穿于政府工作的各个领域、各个环节，不断提高运用法治思维和法治方式解决问题的能力。健全完善重大行政决策公众参与、专家论证、风险评估等制度，提高政府决策的科学化、民主化、法制化水平。完善政府法律顾问制度，逐步实现法律顾问制度全覆盖。自觉接受人大法律监督和政协的民主监督。完善和加强政府内部监督，保障和支持审计、监察等部门依法独立行使监督权。

坚持把作风建设贯穿始终。进一步巩固群众路线教育实践活动成果，坚持不懈抓好“四风”整改。深入推进政府效能建设，切实以治庸提能力、以治懒增效率、以治散正风气。建立政务信息全公开工作机制，加快推进电子政务，积极推行电视问政、网络问政、微博问政，提高政府服务质量和行政效率。多办顺应民意、化解民忧、为民谋利的实事、好事，努力做到群众有呼声，政府有回应，真正以干部的辛苦指数换取群众的幸福指数。

坚持把廉洁从政贯穿始终。严格执行廉洁自律和“八项规定”等制度，全面履行“一岗双责”责任制。守纪律、讲规矩，严格规范权力运行，努力做到集干净干事于一身、廉政勤政于一体。强化行政监察和审计监督，加强对财政资金使用、土地出让、工程招投标、政府采购等重点领域监管。严控“三公”经费和一般性支出，将有限的财力集中到惠民生、促发展上。

各位代表，新形势、新任务，要求我们必须勇于担当、主动作为。让我们高举中国特色社会主义伟大旗帜，在市委的坚强领导下，抢抓机遇、顺势而为，坚定信心、迎难而上，为建设“开放、富裕、和谐、美丽、智慧”新吴忠、与全国全区同步进入全面小康社会而努力奋斗！
